# Diversity and Host Relationships of the Mycoparasite *Sepedonium* (Hypocreales, Ascomycota) in Temperate Central Chile

**DOI:** 10.3390/microorganisms9112261

**Published:** 2021-10-30

**Authors:** Josefa Binimelis-Salazar, Angélica Casanova-Katny, Norbert Arnold, Celia A. Lima, Heraldo V. Norambuena, Gerardo González-Rocha, Götz Palfner

**Affiliations:** 1 Laboratorio de Micología y Micorriza, Departamento de Botánica, Facultad de Ciencias Naturales y Oceanográficas, Universidad de Concepción, Concepción 4030000, Chile; binimelis89@gmail.com; 2 Laboratorio de Ecofisiología Vegetal y Cambio Climático, Departamento de Ciencias Veterinarias y Salud Pública, Universidad Católica de Temuco, Campus Luis Rivas del Canto, Temuco 4780000, Chile; mcasanova@uct.cl; 3 Núcleo de Estudios Ambientales (NEA), Facultad de Recursos Naturales, Universidad Católica de Temuco, Temuco 4780000, Chile; 4 Research Group Natural Products & Metabolomics, Department Bioorganic Chemistry, Leibniz Institute for Plant Biochemistry, 06120 Halle/Saale, Germany; narnold@ipb-halle.de; 5 Laboratorio de Investigación en Agentes Antibacterianos, Facultad de Ciencias Biológicas, Universidad de Concepción, Concepción 4030000, Chile; celia_de_lima@yahoo.com (C.A.L.); ggonzal@udec.cl (G.G.-R.); 6 Departamento de Salud Publica y Prevención, Facultad de Odontología, Universidad de Concepción, Concepción 4030000, Chile; 7 Centro Bahía Lomas, Facultad de Ciencias, Universidad Santo Tomás, Concepción 4030000, Chile; buteonis@gmail.com

**Keywords:** *Sepedonium*, South America, Boletales, endemism

## Abstract

We present the first major survey of regional diversity, distribution and host-association of *Sepedonium*. Whereas the rather scarce worldwide records of this mycoparasitic fungus suggested no specific distribution pattern of most species before, we provide new evidence of endemic and specific host-parasite guilds of *Sepedonium* in Southern South America, including the description of a new species. The corresponding inventory was performed in temperate central Chile. The regional landscape, a mosaic of exotic timber plantations and remnants of native *Nothofagus* forests, facilitates a unique combination of endemic and adventitious Boletales hosts. During a two-year survey, 35 *Sepedonium* strains were isolated and cultured from infected basidiomata of allochthonous *Chalciporus piperatus*, *Paxillus involutus*, *Rhizopogon* spp. and *Suillus* spp., as well as from the native *Boletus loyita*, *B. loyo*, *B. putidus* and *Gastroboletus valdivianus*. Taxonomic diagnosis included morphology of conidia and conidiophores, sequences of ITS, RPB2 and EF1 molecular markers and characteristics of in vitro cultures. Phylogenetic reconstructions were performed using Bayesian methods. Four *Sepedonium* species could be identified and characterized, viz.: *S. ampullosporum*, *S. chrysospermum*, *S. laevigatum* and the newly described species *S. loyorum*. The most frequent species on introduced Boletales was *S. ampullosporum*, followed by *S. chrysospermum* and *S. laevigatum*. *S. loyorum* sp. nov. was found exclusively on native boletacean hosts, separated from its closest relative *S. chalcipori* by micromorphological and molecular attributes. Species descriptions and identification keys are provided. Ecological and biogeographical aspects of endemic and allochthonous symbiotic units consisting of mycoparasite, ectomycorrhizal fungal host and respective mycorrhizal tree are discussed.

## 1. Introduction

Mycophilic fungi belonging to the anamorphic genus *Sepedonium* Link (Hypocreaceae, Ascomycota), teleomorph *Hypomyces* (Fr.) Tul. & C. Tul., are known as highly specialized parasites on basidiomata of various genera of Boletales E.-J. Gilbert [1]. Once infected by *Sepedonium* spp., host structures are hardly ever visibly colonized by other parasitic microorganisms, not even by those known to be also associated to Boletales [2]; this evident exclusion of competitors can be correlated with an arsenal of powerful antibiotic compounds like oligopeptides [3,4] and secondary metabolites [5], produced by the mycoparasite. The type species *Sepedonium chrysospermum* (Bull.) Fr. is characterized by two types of conidia, viz.: ellipsoid, colorless, smooth-walled phialoconidia and globose, yellow aleurioconidia with a coarsely verrucose spore wall, both formed on verticillate conidiophores [6,7]. So far, eight taxa of *Sepedonium* have been recognized [8]: *S. ampullosporum* Damon, *S. brunneum* Peck, *S. chalcipori* Helfer, *S. chlorinum* (Tul. & C. Tul.) Damon, *S. chrysospermum*, *S. laevigatum* Sahr & Ammer, *S. microspermum* Besl and *S. tulasneanum* (Plowr.) Sacc. The main diagnostic attributes have been shape, size and ornamentation of conidia, architecture of conidiophores and in vitro culture characteristics. These morphological features are relatively few, not always discrete and most of them require the examination of live cultures, which may explain the low number of newly described species during most of the 20th century [6,7,8,9]. More recently, DNA-sequence-based phylogeny has proven a valuable tool to detect and separate new species like *S. laevigatum* [3,8,10]. After important taxonomic contributions by [6,8] carried out the most complete study so far on the diversity of *Sepedonium* species, using 52 strains from disjunct localities across different continents and countries, examining their pigment chemistry, host specificity, in vitro growth characteristics and ITS molecular markers.

The host order Boletales (Agaricomycetidae, Basidiomycota), including many ectomycorrhizal species, is represented in most forest ecosystems around the world, but is still poorly studied in extensive geographic regions such as the Neotropics and the Palaeotropics where they reach remarkable divergence [11]. The most common host genera for *Sepedonium* within the families Boletaceae, Paxillaceae, Suillaceae, Sclerodermataceae and Rhizopogonaceae are *Boletus* s.l., *Xerocomus* s.l. and *Paxillus*, among others, whose infected basidiomata typically exhibit large amounts of the characteristic yellow aleurioconidia [1] as visual evidence of the presence of the mycoparasite.

*Sepedonium* spp. have been reported from most continents and biomes where compatible Boletales hosts are present; however, in proportion to the wide range of distribution, available records are scattered and to our knowledge, no extensive regional monitoring of species richness, host diversity and parasite-host specificity, especially in the Southern hemisphere, has been published to date. The fact that most records are distributed in the Northern Hemisphere [8] should be considered due to a historical geographical bias in sampling effort and is contrasted by the first finding of *S. ampullosporum* from Brazil [9]; other species were also sporadically accounted for at higher Southern latitudes, such as *S. chalcipori* in New Zealand [8] and *S.* cf. *chrysospermum* in Chile [12,13]. The first record of the previously undescribed taxon *S*. aff. *chalcipori* (here formally described as *S. loyorum* sp. nov.) on the endemic host *Boletus loyo* Philippi in Chile [3] was a strong incentive for further research on distribution and host-specificity of *Sepedonium* in the South American *Nothofagus* area.

In Chile, the biota of the transition zone between Mediterranean and temperate climate approximately between 35° and 40° S.L., presents an extraordinarily high level of endemism [14,15,16,17,18,19,20]. At the same time this region has been subject to dramatic land use changes driven by extensive agricultural and silvicultural activities, so that the managed and natural woodlands in Central Southern Chile nowadays form a mosaic of often large-scale timber plantations of exotic *Pinus radiata* D. Don or *Eucalyptus* spp. and scattered remnants of native forests of varying size and state of conservation. *Nothofagus* s.l. Blume is the only strictly ectomycorrhizal native tree genus in the Andean-Patagonian forests and hence the only host for endemic ectomycorrhizal fungi in the region [21]. These mixed habitats are supporting a unique combination of autochthonous and adventitious Boletales [22,23,24,25], many of them compatible with mycophilic *Sepedonium* spp., conditions which allowed us a broad approach towards novel knowledge of diversity, distribution and specificity patterns of the mycoparasite and its hosts in a relatively small geographic area.

As the result of a two-year inventory between 2017 and 2018, we provide the first comprehensive study of diversity of *Sepedonium* spp. in temperate central Chile, combining morphological diagnostic attributes, culture characteristics, host diversity and molecular phylogeny based on three DNA markers, viz.: ITS, EF1-α and RPB2. A total of four species was recorded, characterized and identified along with their hosts, one of them new to science. 

## 2. Materials and Methods

### 2.1. Study Area and Material Collection

**Figure 1:**
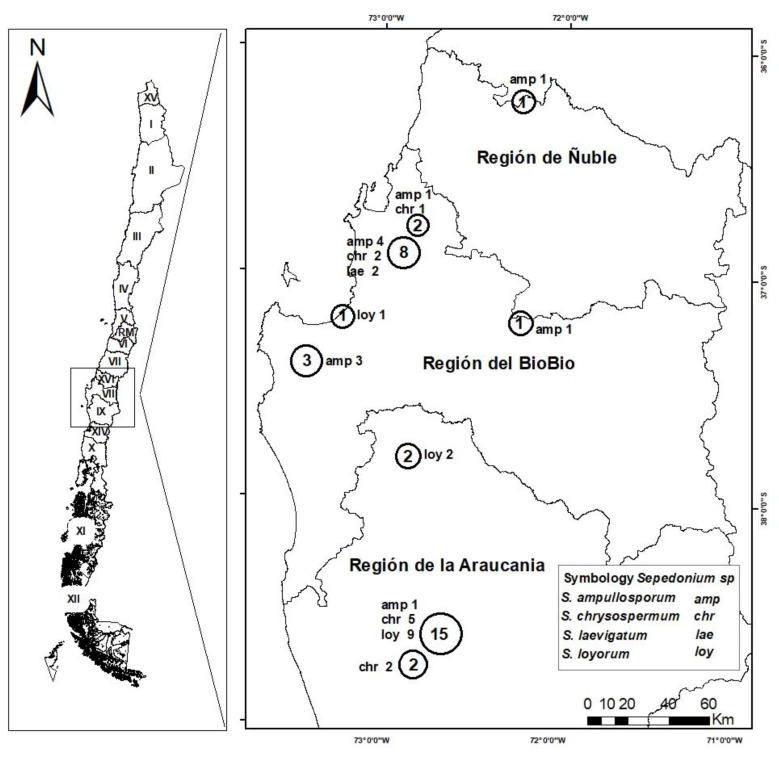
Distribution map of *Sepedonium* spp. collections between 2017 and 2018 in Central Southern Chile.

Basidiomata infected by *Sepedonium* were collected during opportunistic forays in temperate central Chile in the administrative regions of Biobío, Ñuble and Araucanía (Figure 1, Table S1), approximately between 36°15′ and 38°45′ S.L, during the complete mushroom growing season 2017 (April to October) and autumn 2018 (February to May). 

The visited habitats include native Nothofagus forests as well as timber plantations of introduced Pinus radiata and other anthropogenic habitats such as parks and gardens with presence of ectomycorrhizal trees compatible with the requested Boletales hosts. Spotted basidiomata with visible symptoms of infection by Sepedonium were documented in situ, extracted, stored in plastic containers and transferred to the laboratory within the same day where they were refrigerated at 4 °C for further processing. 

### 2.2. Host Species

Basidiomata of Boletales hosts were identified in the field and in the laboratory by macroscopic and microscopic examination of diagnostic morphological attributes, according to keys and reference descriptions [13,22,26] for native Chilean species; for adventitious taxa we consulted [27,28,29], as well as various electronic resources. Dehydrated voucher specimens were deposited at CONC-F (Fungarium G. Palfner, Universidad de Concepción, Concepción, Chile).

### 2.3. Strain Cultivation

Scrapings of *Sepedonium* mycelium and conidia were taken from fresh, infected basidiomata under sterile conditions and placed on Petri dishes with malt peptone extract agar [3]. Three replicates of each strain were incubated at 25 °C and purified from contamination, if necessary, until obtaining axenic cultures. Strains are kept at Laboratorio de Investigación en Agentes Antibacterianos, Universidad de Concepción (Concepción, Chile) and at the Kulturen Sammlung Halle (KSH), Institute for Plant Biochemistry (Halle (Saale), Germany).

### 2.4. Taxonomic Analysis

#### 2.4.1. Optical Microscopy

Pieces of mycelium and conidia scrapings were mounted on glass slides with a drop of distilled water without staining. Specimens were observed at 1000× magnification under an Olympus CX31 compound microscope (Olympus, Tokyo, Japan) equipped with an attached digital camera and drawing device. Diagnostic structures such as conidiophores, phialoconidia and aleurioconidia were examined, measured and documented according to [6].

#### 2.4.2. Scanning Electron Microscopy

Dehydrated samples of *Sepedonium* on infected hosts and from axenic cultures were fixed on SEM mounts with patches of double-sided adhesive tape, metallized with gold and observed in a JSM-6380LV scanning electron microscope (JEOL, Tokyo, Japan), in order to obtain high resolution images of aleurioconidia wall structure.

#### 2.4.3. DNA Extraction

With a sterile inoculation loop, mycelium samples were scraped with or without agar from culture plates and suspended in a buffer solution provided by FastDNA™ SPIN Kit for Soil (MP Biomedicals, Solon, OH, USA), suitable for the extraction of fungal genomic DNA. The manufacturer’s instructions for DNA extraction and purification were followed, including the mechanical lysis step consisting of two 40 s to 6 m/s cycles on a FastPrep-24™ Classic Instrument high-speed homogenizer (MP Biomedicals, Solon, OH, USA). The purified DNA was stored at −20 °C before further processing.

#### 2.4.4. PCR Amplification and Sequencing

For PCR amplification, 2 µL of the extracted DNA (approximately 10 ng) were used as template in a reaction volume of 10 µL, which also contained KAPA Taq ReadyMix with dye (Kapa Biosystems, Cape Town, South Africa) diluted with sterile nuclease-free water, at a final concentration of 1× (corresponding to a concentration of 0.2 µM dNTPs and 1.5 mM MgCl_2_) and 0.2 µM of each of the primers to be used, viz.: ITS, RPB2, EF1-α [30,31,32]. The ITS region was amplified using the primers ITS1: 5′-TCCGTAGGTGAACCTGCGG, and ITS4: 5′-TCCTCCGCTTATTGATATGC. The molecular marker EF1-α was amplified with the following primers EF1T: 5′-ATGGGTAAGGARGACAAGAC, and 1567R: 5′-ACHGTRCCRATACCACCSATCTT). RPB2 was amplified using fRPB2-5F: 5′-GAYGAYMGWGATCAYTTYGG and fRPB2-7cR: 5′-CCCATRGCTTGTYYRCCCAT. The amplification was performed in a Veriti 96 well Thermal Cycler (Applied Biosystems, Waltham, MA, USA) using the following program: 95 °C for 3 min; 35 cycles at 95 °C for 15 s, 54 °C for 15 s and 72 °C for 30 s; a final incubation step at 72 °C for 7 min. The PCR protocol used was the same for all three markers. PCR products were sequenced in one direction (forward) by automatic sequencing at Macrogen (ABI3730XL, Seoul, Korea). 

#### 2.4.5. Sequence Processing and Phylogenetic Analysis

The sequences obtained from Macrogen for each molecular marker (ITS1, EF1-α, RPB2) were edited through the Codon Code Aligner v. 3.0.3 program (CodonCode Corporation, www.codoncode.com, accessed on 1 June 2021). The sequences were aligned using the MUSCLE program [33] also integrating *Sepedonium* sequences available from the GenBank database (Table S2). The saturation test was performed in the DAMBE v. 5.2 program [34], to evaluate the usefulness of the sequences for phylogenetic analyses. The proportion of invariant sites, a key parameter for the saturation test, was obtained with jModeltest 2 [35]; the same software was used to identify the best fit nucleotide replacement model for each gene which turned out T93 + G for ITS1, EF1- α and RPB2 under Akaike’s reporting criteria.

Two species trees were recovered in *BEAST, a component of BEAST v. 2.3.2 [36], one performed at the individual level using the ITS 1 molecular marker (68 strains) and the other performed at the species level using 25 individuals (eight strains of *S. ampullosporum*; four strains of *S. chrysospermum*; seven strains of *S. loyorum*; four strains of *S. chalcipori* and two strains of *S. laevigatum*) for which the ITS1, EF1-α and RPB2 sequences were available (Table S2). This second phylogenetic tree was built under the coalescent multispecies model [37,38]. We used the same nucleotide substitution and model configuration for each dataset. Considering that T93 + G is a simple evolutionary model, we evaluated the effect of more complex models (GTR + G + I), obtaining identical topologies. For each dataset, 100 million iterations were performed and sampled every 1000 steps, the first 25% of the results being discarded by burn-in. The convergence of MCMC analysis was visually examined in Tracer v1.6 [39] to verify stationarity and effective sample sizes (ESS) greater than 200. For all analyses *Trichoderma aerugineum* Jaklitsch was used as external group. Graphic design of the trees was done with the Fig Tree v.1.4.3 program [40]. We also calculated uncorrected pairwise genetic distances based on ITS in MEGA 5.0.

## 3. Results

### 3.1. Taxonomy of Sepedonium Strains and Host Taxa

The 35 *Sepedonium* strains sucessfully retrieved from different locations and hosts (Figure 1, Table S1), apart from molecular analysis, were characterized and identified based on diagnostic micromorphological attributes, viz.: phialoconidiophore branching, phialid length, size and shape of phialoconidia, size and ornamentation of aleurioconidia.

Identified species are *Sepedonium ampullosporum*, *S. chrysospermum*, *S. laevigatum* and *S. loyorum* sp. nov. There is evidence for a non-random parasite-host association for the study area, given that the six allochthonous hosts (*Chalciporus piperatus* (Bull.) Bataille, *Paxillus involutus* (Batsch) Fr., *Rhizopogon luteolus* Fr., *Rh. roseolus* (Corda) Th. Fr., *Suillus granulatus* (L.) Roussel, *S. luteus* (L.) Roussel) were infected by *Sepedonium ampullosporum*, *S. chrysospermum* and *S. laevigatum*, while the four native hosts (*Boletus loyita* E. Horak, *B. loyo* Philippi, *B. putidus* E. Horak, *Gastroboletus valdivianus* E. Horak) were infected exclusively by *Sepedonium loyorum* sp. nov.

### 3.2. Phylogenetic Characterization of Sepedonium Strains

We obtained 64 sequences with 517 base pairs for the ITS1 marker, 25 sequences with 659 base pairs for the RPB2 gene and 25 sequences with 596 base pairs for the EF1-α gene (Table S2). All three markers showed low saturation with *p* < 0.05 (ITS1: 0.33971; EF1-α: 0.00188; RPB2: 0.54632). The polymorphic sites for the ITS1 marker are 70, 224 for the EF1-α gene and 156 for the RPB2 gene.

**Figure 2:**
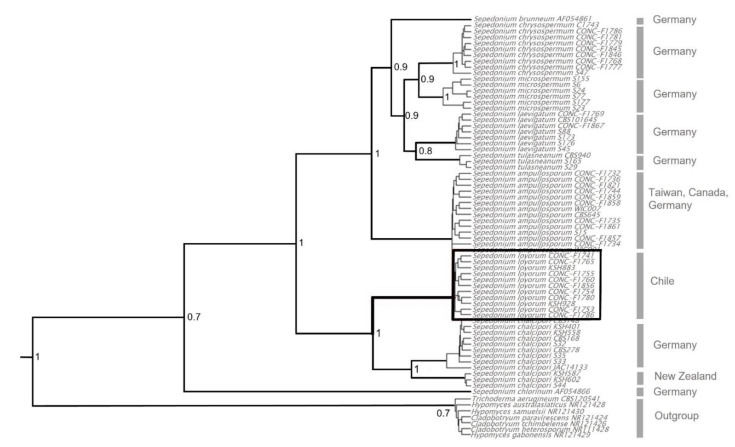
Phylogenetic tree reconstructed using the Beast 2.0 program showing kinship relationship of *Sepedonium loyorum* sp. nov. with all other known species, inferred from the internal transcribed spacer region of the ITS genes (ITS 1); outgroup is represented by *Trichoderma aerugineum*, *Hypomyces* spp. and *Cladobotryum* spp.; the values on the nodes correspond to the posteriori probability.

For the tree represented by the ITS molecular marker (Figure 2), the genus *Sepedonium* was recovered as monophyletic (1.0 Posterior Probability) by Bayesian inference analysis. The nine different taxa, including the four species known from Chile, are divided in three main clades: The first clade is represented by *Sepedonium chlorinum* which is supported by 0.71 PP (posteriori probability). The second clade is composed of *S. loyorum* sp. nov. and *S. chalcipori* and is supported by 0.98 PP. The third clade includes *S. ampullosporum*, *S. chrysospermum*, *S. microspermum*, *S. tulasneanum*, *S. laevigatum* and *S. brunneum*, which is supported by 1.0 PP.

**Figure 3:**
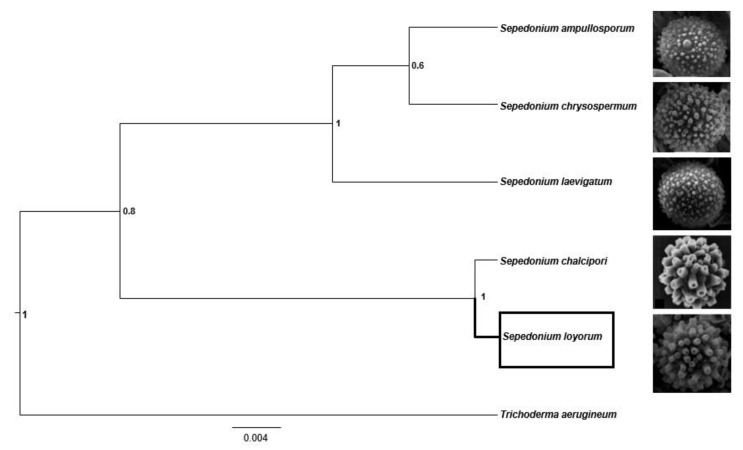
Phylogenetic species tree reconstructed using Beast 2.0 program showing *Sepedonium* species delimitation with *Trichoderma aerugineum* as external group, inferred from ITS, RPB2 and EF1-α-multigene sequence alignment; node values express posterior probability; aleurioconidia micrographs obtained from this study except *Sepedonium chalcipori* micrograph extracted and modified from [8].

The multilocus species tree (ITS, EF1 and RPB2), including the four taxa reported from Chile and *S. chalcipori* as neighbor taxon of *S. loyorum* (Figure 3), recovered the *Sepedonium* species cluster as monophyletic (PP = 1.0). The species fall into two major clades (PP = 0.7), one represented by *S. loyorum* sp. nov. and *S. chalcipori* with a PP support = 1.0, the second clade including *S. ampullosporum*, *S. chrysospermum* and *S. laevigatum* supported with PP = 1.0 (Figure 3).

The uncorrected pairwise genetic distances based on ITS showed that *S. chalcipori* has 3% of genetic distance with respect to *S.* *loyorum* sp. nov.

### 3.3. Morphological Characterization of Sepedonium spp.

**Figure 4:**
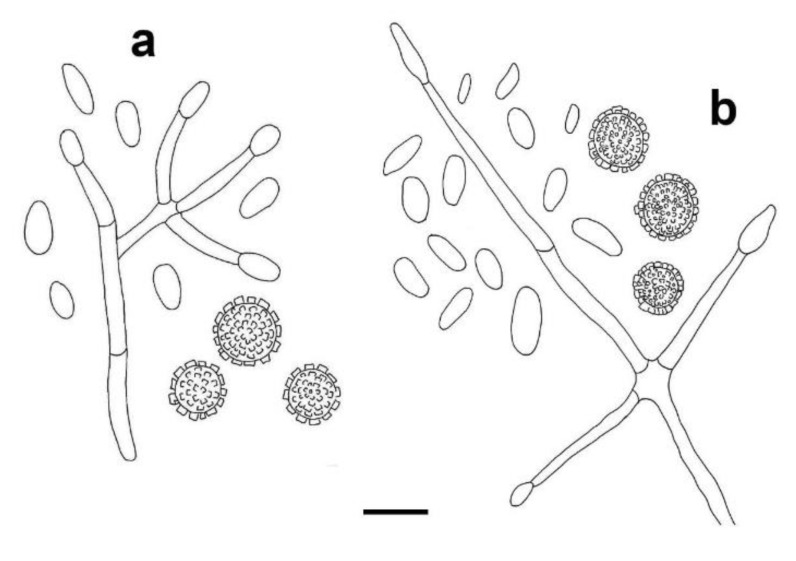

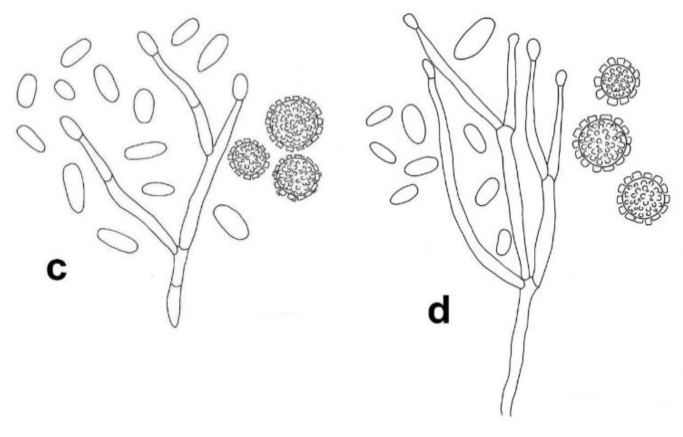
*Sepedonium loyorum* sp. nov., conidiophores, phialoconidia and aleurioconidia in axenic culture (MPA) obtained from four different host species; (**a**) from *Boletus loyita*, strain CONC-F1760; (**b**) from *B. loyo*, strain CONC-F1755; (**c**) from *B. putidus*, strain CONC-F1754 (holotype); (**d**) from *Gastroboletus valdivianus*, strain CONC-F1741; bar 20 µm.

**Figure 5:**
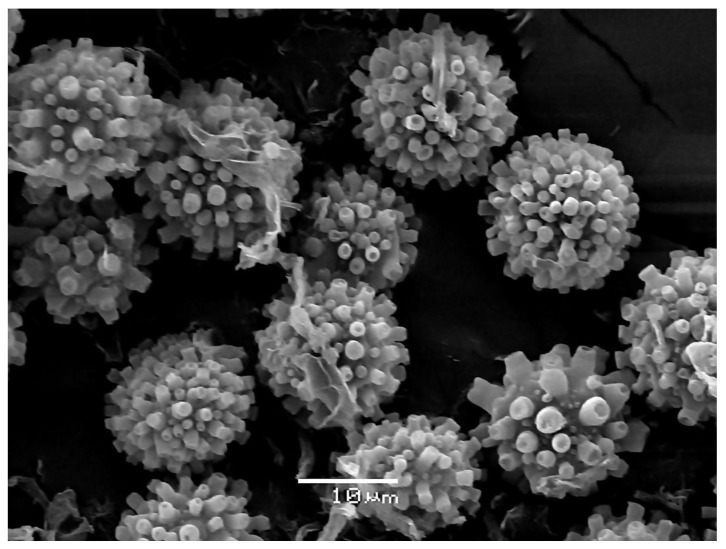
*Sepedonium loyorum* sp. nov. (CONC-F1754, holotype), SEM micrograph of aleurioconidia.

**Figure 6:**
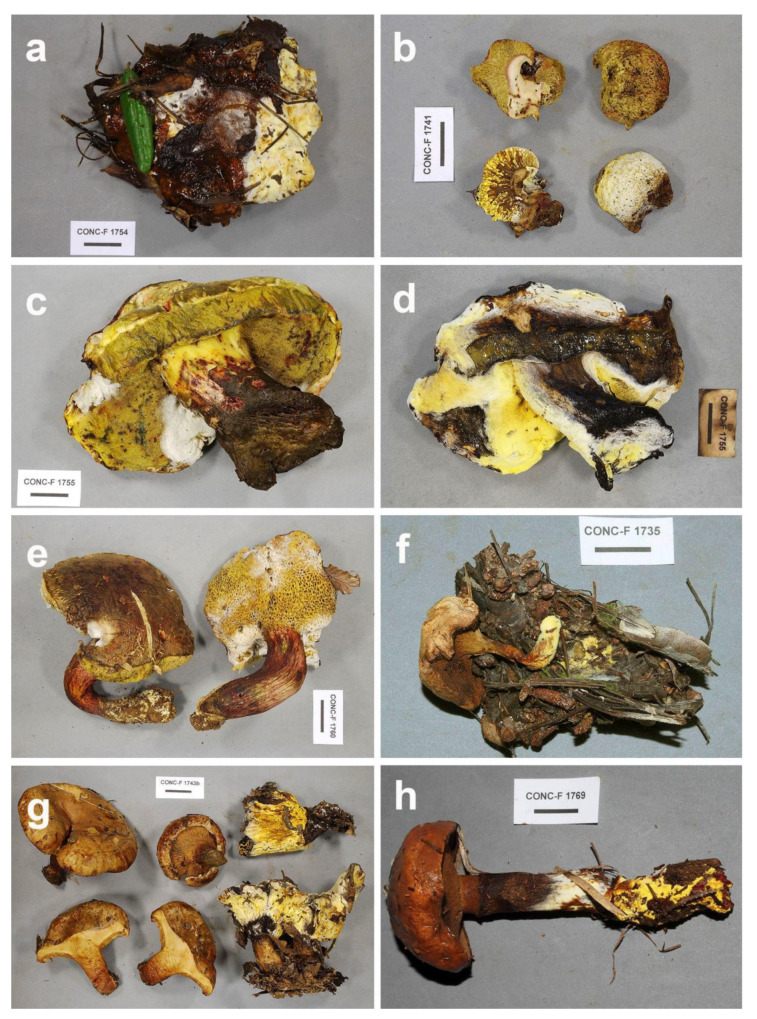
*Sepedonium* spp. from central temperate Chile on selected hosts; (**a**) *S. loyorum* sp. nov. on *Boletus putidus*, CONC.F1754 (holotype); (**b**) *S. loyorum* on *Gastroboletus valdivianus*, CONC.F1741; (**c**) *S. loyorum* on *B. loyo*, CONC-F1755; (**d**) same sample as c after one week storage at 4 °C; (**e**) *S. loyorum* on *B. loyita*, CONC-F1760; (**f**) *S. ampullosporum* on *Chalciporus piperatus*; (**g**) *S. chrysospermum* on *Paxillus involutus*, CONC-F1743; (**h**) *S. laevigatum* on *Suillus luteus*, CONC-F1769; bar in all Figures: 2 cm.

**Figure 7:**
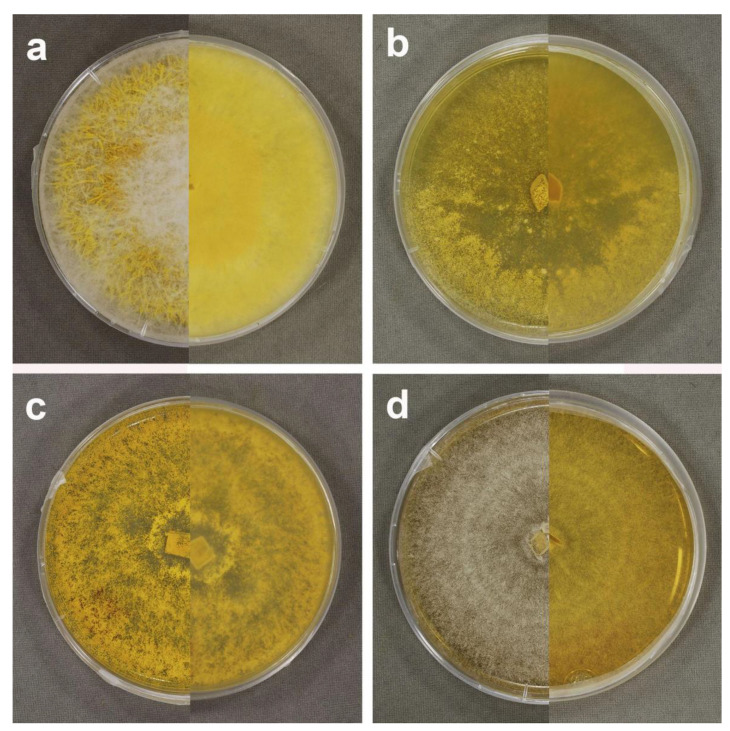
In vitro cultures of *Sepedonium* spp. from temperate central Chile after two weeks growth on MPA in 90 mm Petri dishes at 20–25 °C, left half of each photo showing upper face, right half showing reverse of respective culture dish; (**a**) *S. ampullosporum* from *Paxillus involutus*, CONC-F1732; (**b**) *S. chrysospermum* from *Paxillus involutus*, CONC-F1743; (**c**) *S. laevigatum* from *Suillus luteus*, CONC-F1769; (**d**) *S. loyorum* sp. nov. from *Boletus putidus*, CONC-F1754 (holotype).

**Figure 8:**
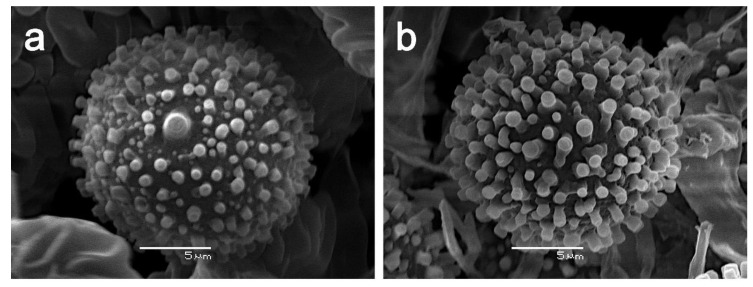

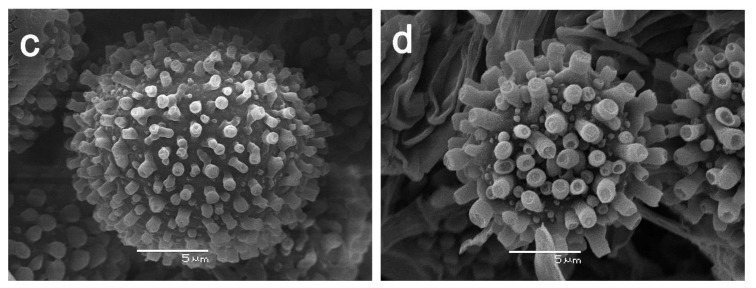
Scanning electron micrographs of aleurioconidia of Chilean *Sepedonium* spp.; (**a**) *S. ampullosporum* CONC-F 1821; (**b**) *S. chrysospermum* CONC-F 1743; (**c**) *S. laevigatum* CONC-F 1769; (**d**) *S. loyorum* CONC-F 1741.

*Sepedonium loyorum* Palfner, Casanova-Katny, N. Arnold & Binimelis-Salazar sp. nov. (Figure 4, Figure 5, Figure 6a–e, Figure 7d and Figure 8d).

*Etymology*: loyorum: latinized plural of loyo, referring to the main host species *Boletus loyo* and *Boletus loyita*; both epithets are derived from the corresponding native Chilean (mapuzungún) names which are “Loyo” for *B. loyo* and “Pichiloyo” (small loyo) for *B. loyita*. 

*Diagnosis*: Tegumentum tomentosum, in sporocarpiis boletorum Chilensium, primo album, maturitate aureum; phialoconidiophori in cultivo (MPA) verticillati, 2–3 (4) phialidibus curtibus, 23–60 (80) × 2–3.5 µm; phialoconidia incolorata, tenuitunicata, subcylindracea, ovoidea vel ellipsoidea, magnitudine variabilis, 8–15 × 3–7 µm; aleurioconidia in hyphis lateralibus, primo incolorata, deinde lutea, crassitunicata, globosa, 10–15 µm diam, verrucosa; verrucae breviter cylindraceae vel doliiformae, 1–3 µm altae, apice paulum depresso; coloniae in MPA 25 °C post septem dies 25–30 mm diametro, paucim mycelio aerio albo, medium nutricium lutescens.

Mycelium forming a tomentose to pubescent layer or mat on carpophores of native Chilean Boletaceae, white in the beginning, at maturity golden yellow (Figure 6a–e); conidiophores (Figure 4) in culture (MPA) hyaline, verticillate, septate, verticills bearing 2–3(4) short phialides, 23–60 (80) × 2–3.5 µm; phialoconidia (Figure 4) colorless, thin-walled, ovoid, ellipsoid or subcylindrical, rather variable in size, 8–15 × 3–7 µm; aleurioconidia (Figure 4, Figure 5 and Figure 8d) colorless in the beginning, then becoming yellow, thick- walled, globose, 10–15 µm in diam, verrucose, warts cylindrical to barrel- volcano- or amphora-shaped, 1–3 µm long, typically with apical depression, densely arranged; colonies (Figure 7d) slowly growing in MPA, 25–30 mm in seven days at 25 °C, forming a thin whitish, rather compact mat of tomentose mycelium, only slowly becoming yellow by mature aleurioconidia, culture medium turning yellow; teleomorph not known. 

*Hosts*: *Boletus loyita* E. Horak, *Boletus loyo* (Philippi) Speg., *Boletus putidus* E. Horak, *Gastroboletus valdivianus* E. Horak.s

*Examined Material*: Holotype: Chile, Región de la Araucanía, Temuco, Reserva Rucamanque, on *Boletus putidus*, 12 April 2017, leg. A. Casanova-Katny (CONC-F1754, KSH1001); Further material: Chile, Región de la Araucanía, Temuco, Reserva Rucamanque, on *Boletus putidus*, 30 March 2017, leg. A. Casanova-Katny (CONC-F1741); Chile, Región de la Araucanía, Temuco, Reserva Rucamanque, on *Boletus putidus*, 12 April 2017, leg. A. Casanova-Katny (CONC-F1752); Chile, Región de la Araucanía, Temuco, Reserva Rucamanque, on *Boletus loyo*, 12 April 2017, leg. A. Casanova-Katny (CONC-F1753); Chile, Región de la Araucanía, Temuco, Reserva Rucamanque, on *Boletus loyo*, 12 April 2017, leg. A. Casanova-Katny (CONC-F1755); Chile, Región del Biobío, Santa Juana, on *Boletus putidus*, 18 April 2017, leg. C. Lima (CONC-F1756); Chile, Región de la Araucanía, Temuco, Reserva Rucamanque, on *Gastroboletus valdivianus*, 27 April 2017, leg. A. Casanova-Katny (CONC-F1758); Chile, Región de la Araucanía, Temuco, Reserva Rucamanque, on *Boletus loyita*, 27 April 2017, leg. A. Casanova-Katny (CONC-F1760); Chile, Región de la Araucanía, Angol, Nahuelbuta, on *Boletus loyo*, 01 May 2017, leg. N. Arnold (CONC-F1765); Chile, Región de la Araucanía, Angol, Nahuelbuta, on *Boletus loyo*, 1 May 2017, leg. N. Arnold (CONC-F1766); Chile, Región de la Araucanía, Temuco, Reserva Rucamanque, on *Boletus loyita*, 09 May 2017, leg. A. Casanova-Katny (CONC-F1780); Chile, Región de la Araucanía, Temuco, Reserva Rucamanque, on *Gastroboletus valdivianus*, 24 April 2018, leg. A. Casanova-Katny (CONC-F1856). *Comment*: According to our results, *Sepedonium* aff. *chalcipori* cited by [3] is conspecific with *S. loyorum*.

*Sepedonium ampullosporum* Damon, Mycologia 44(1): 91, 1952 (Figure 6f, Figure 7a and Figure 8a).

Conidiophores in culture (MPA) hyaline, verticillate, septate, verticills bearing up to 3 phialides, 43–128 × 3–5 µm; phialoconidia hyaline, lecythiform, 15–22 × 5–8 µm; aleurioconidia (Figure 8a) yellow, thick- walled, globose, 14–19 (21) µm in diam., verrucose, with short conical or subcylindrical warts, 1–1.5 µm long; colonies in MPA (Figure 7a) first white and forming abundant aerial mycelium with cottony or stringy texture, soon becoming yellow, culture medium turning yellow; growth rate 5.2 mm/day in an average of 7 days at 25 °C; no differences of micromorphological structures were found between colonized hosts and axenic culture.

*Hosts*: *Suillus luteus* (L.) Roussel 1796, *Suillus granulatus* (L.) Roussel 1796, *Chalciporus piperatus* (Bull.) Bataille 1908, *Paxillus involutus* (Batsch) Fr. 1838, *Rhizopogon luteolus* Fr. 1817.

*Examined Material*: Chile, Región del Biobío, Concepción, Puchacay, on *Paxillus involutus*, 4 March 2017, leg. G. Palfner (CONC-F1732); Chile, Región del Biobío, Concepción, Reserva Nonguén, on *Suillus luteus*, 28 March 2017, leg. A. Casanova-Katny (CONC-F1734); Chile, Región del Biobío, Concepción, Reserva Nonguén, on *Chalciporus piperatus*, 28 March 2017, leg. A. Casanova-Katny (CONC-F 1735); Chile, Región del Biobío, Concepción, Reserva Nonguén, on *Suillus luteus*, 28 March 2017, leg. A. Casanova-Katny (CONC-F1736); Chile, Región del Biobío, Concepción, Reserva Nonguén, on *Suillus luteus*, 4 April 2017, leg. A. Casanova-Katny (CONC-F1744); Chile, Región del Biobío, Concepción, Reserva Nonguén, on *Suillus luteus*, 4 May 2017, leg. G. Palfner (CONC-F1767); Chile, Región del Biobío, Arauco, on *Suillus granulatus*, 19 October 2017, leg. G. Palfner (CONC-F1821); Chile, Región de Ñuble, Quirihue, on *Suillus luteus*, 29 April 2018, leg. N. Arnold (CONC-F1857); Chile, Región del Biobío, Arauco, Curanilahue, on *Rhizopogon luteolus*, 10 May 2018, leg. S. Troncoso (CONC-F1858); Chile, Región del Biobío, Arauco, Curanilahue on *Suillus luteus*, 10 May 2018, leg. S. Troncoso (CONC-F1859); Chile, Región del Biobío, Arauco, Curanilahue, on *Suillus luteus*, 11 May 2018, S. Troncoso (CONC-F1861); Chile, Región de la Araucanía, Temuco, Reserva Rucamanque, on *Suillus luteus*, 10 May 2018, leg. A. Casanova-Katny (CONC-F1869).

*Sepedonium chrysospermum* (Bull.) Fr., Syst. Mycol. (Lundae) 3 (2): 438, 1832 (Figure 6g, Figure 7b and Figure 8b).

Conidiophores in culture (MPA) hyaline, verticillate, septate, verticills bearing up to 3 phialides, 55–110 × 4.5–6 µm; phialoconidia hyaline, ellipsoid, 6–24 × 3–11 µm; aleurioconidia (Figure 8c) yellow, thick- walled, globose, 14 (16)–19 (22) µm in diam., verrucose with short cylindrical warts 1–1.5 µm long; colonies (Figure 7b) in MPA golden yellow, surface smooth to grainy, culture medium turning yellow, growth rate 4.6 mm/day in an average of 7 days at 25 °C; no differences in micromorphological structures were found between colonized host and axenic culture.

*Examined Material*: Chile, Región de la Araucanía, Temuco, Universidad Católica de Temuco, on *Paxillus involutus*, 29 March 2017, leg. A. Casanova-Katny (CONC-F1743b); Chile, Región del Biobío, Concepción, Reserva Nonguén, on *Suillus luteus*, 4 May 2017, leg. N. Arnold (CONC-F1768); Chile, Región de la Araucanía, Temuco, Reserva Rucamanque, on *Paxillus involutus*, 9 May 2017, leg. A. Casanova-Katny (CONC-F1777); Chile, Región de la Araucanía, Temuco, Reserva Rucamanque, on *Paxillus involutus*, 9 May 2017, leg. A. Casanova-Katny (CONC-F 1778); Chile, Región de la Araucanía, Temuco, Cerro Ñielol, on *Paxillus involutus*, 9 May 2017, leg. N. Arnold (CONC-F1779); Chile, Región de la Araucanía, Curacautín, Andenrose lodge, on *Paxillus involutus*, 5 May 2017, leg. N. Arnold (CONC-F1781); Chile, Región del Biobío, Concepción, Reserva Nonguén, on *Paxillus invoutus*, 16 May 2017, leg. A. Casanova-Katny (CONC-F1786); Chile, Región de la Araucanía, Temuco, Reserva Rucamanque, on *Paxillus involutus*, 28 Februery 2018, leg. A. Casanova-Katny (CONC-F1845); Chile, Región de la Araucanía, Temuco, Reserva Rucamanque, on *Paxillus involutus*, 28 February 2018, leg. A. Casanova-Katny (CONC-F1846).

*Sepedonium laevigatum* Sahr & Ammer, Mycologia 91 (6): 939, 1999 (Figure 6h, Figure 7c and Figure 8c). 

Conidiophores in culture (MPA) hyaline, verticillate, septate, verticills bearing 1–2 long phialides, 115–220 × 3–4.5 µm; phialoconidia hyaline, ellipsoid 8–26 × 3–11 µm; aleurioconidia (Figure 8d) yellow, thick- walled, globose, 13 (14)–17 (19) µm, verrucose with short cylindrical warts 1–1.5 µm long; colonies (Figure 7c) in MPA deep golden yellow to orange yellow, surface smooth to grainy, culture medium turning orange yellow, growth rate 7.5 mm/day in an average of 7 days at 25 °C; no differences of micromorphological structures were found between colonized host and axenic culture.

*Hosts*: *Suillus luteus* (L.) Roussel 1796, *Rhizopogon roseolus* (Corda) Th. Fr. 1909.

*Examined Material*: Chile, Región del Biobío, Concepción, Reserva Nonguén, on *Suillus luteus*, 4 May 2017, leg. G. Palfner (CONC-F 1769); Chile, Región del Biobío, Concepción, Reserva Nonguén, on *Rhizopogon roseolus*, 16 May 2017, leg. A. Casanova-Katny (CONC-F 1867).

### 3.4. Host Specificity and Frequency

Table 1 shows the frequency with which each of the recorded *Sepedonium* spp. was found on the respective host species. 

Whereas total frequency of *S. ampullosporum*, *S. chrysospermum* and *S. loyorum* sp. nov. was similar (31%, 29% and 34%, respectively), *S. laevigatum* with a mere 6% of total records was clearly the rarest species within the survey.

Endemic Boletaceae so far have been found to be exclusively colonized by *S. loyorum* sp. nov. and, correspondingly, the latter has not been registered on an allochtonous host which indicates a highly specific parasite-host association. Among the three widely distributed species, *S. chrysospermum* shows a marked preference for *Paxillus involutus*, with nine out of ten records. The relatively high number of records of *S. ampullosporum* on *Suillus luteus* (six records) is rather owed to the high frequency of the host species in the visited habitats (pine plantations). 

### 3.5. Keys for Sepedonium spp.

The following dichotomous key, apart from the newly described *S. loyorum*, includes all currently accepted *Sepedonium* species treated by [6,7,8] and for which DNA sequences could be retrieved; species recorded in Chile to date are marked with bold letters.
**1** Aleurioconidia subcylindrical, spindle-shaped or ellipsoid, finely warty or longitudinally grooved*2*1* Aleurioconidia globose, covered with prominent warts (sea-mine-shaped)3**2** Aleurioconidia ellipsoid, greyish brown, finely warty to smooth *S. tulasneanum*2* Aleurioconidia subcylindrical to spindle-shaped, yellow, longitudinally grooved*S. chlorinum***3** Phialoconidia lecythiform, in Chile on allochthonous hosts ***S. ampullosporum***3* Phialoconidia oval or ellipsoid, in Chile on allochthonous or native hosts4**4** Phialids rarely over 60 µm long, only exceptionally reaching 100µm54* Few or most phialids over 100 µm long7**5** Mature aleurioconidia 8–14 µm in diam., with angular warts, on *Xerocomus chrysenteron* and related species*S. microspermum*5* Mature aleurioconidia ≥15 µm in diam., warts cylindrical, barrel- or amphora-shaped, on different hosts6**6** Phialids up to 8 per verticill, L/W of phialoconidia up to 3, on *Chalciporus piperatus**S. chalcipori*6* Phialids up to 3 (4) per verticill, L/W of phialoconidia up to 4.3, on native Chilean Boletacean hosts***S. loyorum*****sp. nov.****7** Aleurioconida brownish, on*Suillus pictus S. brunneum*7* Aleurioconidia yellow8**8** Phialids up to 120 µm long, colonies slowly growing, up to 5 mm/day in MPA***S. chrysospermum***8* Phialids up to 250 µm long, colonies fast-growing, up to 8 mm/day in MPA***S. laevigatum***

Supplementary key for Chilean species of *Sepedonium* based on morphology of axenic strains cultivated in vitro (after growing two weeks on MPA in 90 mm Petri dishes at 20–25 °C):
**1** Plate surface after few days covered by abundant and cottony to stringy aerial mycelium***S. ampullosporum***1* Plate surface remaining tomentose to grainy or almost smooth, with little or no aerial mycelium2**2** Colony surface lastingly grayish-white, only slowly turning yellowish***S. loyorum* sp. nov.**2* Colony surface after few days turning intensive golden yellow3**3** Colony fast-growing, reaching the border of the plate after about one week, surface deep golden yellow, becoming almost orange***S. laevigatum***3* Colony slow-growing, reaching the border of the plate after about two weeks***S. chrysospermum***

## 4. Discussion

### 4.1. Regional Diversity and Frequency of Sepedonium spp.

During our survey of mycophilic *Sepedonium* in temperate central Chile, four species could be retrieved from Boletales hosts growing in woody vegetation, and be characterized and identified by morphological and molecular diagnostic attributes: The newly described *S. loyorum* parasites regionally endemic Boletaceae, viz.: *Boletus loyita*, *B. loyo*, *B. putidus* and *Gastroboletus valdivianus*, whereas *S. ampullosporum*, *S. chrysospermum* and *S. laevigatum* were identified on allochthonous hosts including the Boletales genera *Chalciporus*, *Paxillus*, *Rhizopogon* and *Suillus*. *S. ampullosporum* was found to be the most frequent taxon, *S. laevigatum* the most rarely encountered species and *S. chrysospermum*, in concordance with [1,2,3,4,5,6,7,8], showed strong preference for *Paxillus involutus*. The total of identified species accounts for about 44% of all *Sepedonium* spp. globally known to date and hence represents a considerable diversity in a geographically rather limited region.

### 4.2. Phylogenetic Kinship of S. loyorum sp. nov.

Our data indicate that *Sepedonium loyorum* sp. nov. is probably co-endemic with its hosts; it can be distinguished morphologically from its closest phylogenetic relative *S. chalcipori* by architecture and dimensions of phialoconidiophores. Our Bayesian phylogenetic tree based on ITS gene recovered *S. loyorum* sp. nov. as sister species of *S. chalcipori* with high posterior probability (1.0). Unsurprisingly, our analyses resulted in a topology not congruent with the neighbor-joining tree of [8]. The most obvious rearrangement involves *S. brunneum* as sister species of a clade composed by *S. chrysospermum*, *S. microspermum*, *S. laevigatum* and *S. tulasneanum*, with *S. tulasneanum* as sister species of *S. laevigatum*. This pattern is largely coherent with the ITS phylogeny obtained by [41] for seven species of *Sepedonium* in order to elucidate the kinship of *S. microspermum* from Iran.

Our multilocus species tree (based on ITS, RPB2 and EF1-α) also recovered *S. loyorum* sp. nov. as sister species of *S. chalcipori* with high posterior probability (1.0), this result supports the concept of *S. loyorum* sp. nov. being a phylogenetic species different from *S. chalcipori* following the phylogenetic species concept criteria [42].

### 4.3. Biogeographical Aspects of Sepedonium in Chile

This study represents, to our knowledge, the first survey of *Sepedonium* in a major coherent geographical area. Although *S. chrysospermum* has been historically reported rather frequently from several countries on both hemispheres including Chile, not all records may have been correctly identified, especially those dating before the comprehensive studies by [6,7,8]: It should be considered that *S. chrysospermum* was one of only about three species described until the second half of the 20th century, also that aleuroconidia on infected host basidiomata often were the principal or even only diagnostic attribute to be examined, and finally that molecular tools have been applied for species delimitation for only about 30 years before date.

Published worldwide records of other *Sepedonium* spp. are still too scarce and scattered to allow assertions about their biogeography. However, the apparently endemic *S. loyorum* seems to be much more specifically associated to its regional environment and hosts than has been previously assumed for other species of the genus. Due to its evident affiliation to endemic Boletaceae from South American *Nothofagus* forests, a Gondwanean origin of *S. loyorum* appears likely. This suggestion is strengthened by the observation that its closest phylogenetic neighbour, *S. chalcipori*, seems to have a geographical connection to New Zealand [8], although it may have been introduced in Australasia from the northern hemisphere together with its host *Chalciporus* associated with exotic pine species [43]. In any case it would be highly interesting to investigate co-evolution of both species with their known hosts which may also shed new light on diversification and mycogeographical aspects of Boletales on the Southern hemisphere. None of the other three detected *Sepedonium* species (*S. ampullosporum*, *S. chrysospermum*, *S. laevigatum*) could be found on endemic Chilean boletes during our study which allows the conclusion that those taxa, like their respective hosts and associated trees, are adventitious in Chile. The report of *S. chrysospermum* on *B. bresinskyanus* Garrido, an insufficiently known Chilean bolete [13], may be the result of a misidentification based on the historic taxonomic limitations mentioned above.

### 4.4. Sepedonium-Resistant Boletales

An interesting *Sepedonium* host group due to contrasting infection patterns in the studied area is the *Paxillus-Austropaxillus* complex: *Austropaxillus boletinoides* (Singer) Bresinsky & Jarosch and *A. statuum* (Speg.) Bresinsky & Jarosch are common native ectomycorrhizal fungi in Chilean *Nothofagus* forests but, contrasting our expectations, were never found to be infected by *Sepedonium* during our surveys, whereas the adventitious *Paxillus involutus* which commonly grows at disturbed sites like gardens, parks and timber plantations under introduced trees, such as chestnut, oak or eucalypt, but also under native *Nothofagus*, was regularly infected, preferentially by *S. chrysospermum*. Phylogenetic distance between both genera within the Boletales, may be decisive for this difference in susceptibility to the mycoparasite: the genus *Austropaxillus* was separated from *Paxillus* by [44], mainly based on molecular evidence, and convincingly placed in the family Serpulaceae together with *Gymnopaxillus* and *Serpula* [45,46]. As to our knowledge, none of the three genera has so far been reported to be parasitized by *Sepedonium*, another example that host-parasite relationships can be important indicators of phylogeny.

### 4.5. Trophic Connections and Specificity within Parasite-Host Guilds: Invasive vs. Endemic Taxa

Considering that almost all detected Boletales hosts of *Sepedonium* are ectomycorrhizal symbionts, we can contemplate tripartite trophic associations between mycoparasite, mycorrhizal fungal host and mycorrhizal tree which may even share their status of endemism as in the case of *S. loyorum* and its endemic hosts which again are forming mycorrhiza with *Nothofagus* species autochthonous in Southern South America. In the special case of *Chalciporus piperatus* which is supposed to parasitize mycelium of *Amanita muscaria* [43] and which we found infected by *S. ampullosporum*, this association would be still further extended to the epiparasitic level, viz.: *Sepedonium*-*Chalciporus–Amanita* and hence to a quadripartite symbiosis when carbon flux from the mycorrhizal tree is taken into account.

Looking at these rather complex nutrient pathways towards *Sepedonium* in forest ecosystems, it is of importance to estimate the invasive potential of allochthonous parasite-host associations in endemic communities, especially in a mosaic landscape consisting of exotic timber plantations and native forest remnants which characterizes not only our study area in central Chile, but nowadays exists globally in many regions [25,43,47]. *Amanita muscaria* accompanied by *Chalciporus piperatus* was originally adventitious with introduced Monterrey Pine in Southern Chile, but is now observed in pure *Nothofagus* forest with increasing frequency [24,25]; it can therefore safely be assumed that a host-tree switch has taken place from *Pinus* to *Nothofagus* which has the capacity to promote penetration of *Amanita* into native forest as a precursor or vector, as well for *Chalciporus* as for exotic *Sepedonium* spp., where competition with, or even replacement of, endemic fungi on both, mycorrhizal and mycoparasitic level may occur. Time will reveal whether resilience of the endemic taxa is strong enough to integrate this co-invasive force in a stable community, without erosion or even loss of native fungal diversity.

### 4.6. Conservation Criteria for Sepedonium on Threatened Hosts

A documented and pressing threat to endemic Chilean Boletaceae and associated *S. loyorum* is the decrease and deterioration of their native *Nothofagus* forest habitats as a result of direct and indirect effects of land use change in central and Southern Chile [48,49,50]. The conservation category of all four identified hosts has been classified between 2014 and 2019 by IUCN criteria: *Boletus loyo* and *Gastroboletus valdivianus* as threatened (EN), *B. loyita* as vulnerable (VU) and *B. putidus* as nearly threatened (NT) (https://clasificacionespecies.mma.gob.cl/, accessed on 1 June 2021). In their global proposal of conservation strategies for metazoan parasites [51], the authors state that “parasites face a double threat: they are directly vulnerable to extinction due to anthropogenic factors like climate change or invasive species, and indirectly vulnerable through coextinction with hosts, especially in changing environments”. This is certainly also true for mycophilic fungi and, consequently, conservation concepts should be applied for species with endemic status and specific association to threatened hosts and habitats, such as *Sepedonium loyorum*.

## Figures and Tables

**Table 1:** Frequency of mycoparasite-host combinations of *Sepedonium* spp. registered in temperate central Chile (*n* = 35); species in alphabetic order.

	*S. ampullosporum*	*S. laevigatum*	*S. chrysospermum*	*S. loyorum*
Endemic hosts:				
*Boletus loyita*				2
*Boletus loyo*				4
*Boletus putidus*				3
*Gastroboletus valdivianus*				3
Allochthonous hosts:				
*Chalciporus piperatus*	1			
*Paxillus involutus*	2		9	
*Rhizopogon roseolus*		1		
*Rhizopogon luteolus*	1			
*Suillus luteus*	6	1	1	
*Suillus granulatus*	1			
Total:	11 (31%)	2 (6%)	10 (29%)	12 (34%)

## Data Availability

DNA sequence data have been deposited in GenBank, phylogenetic tree information in TreeBase database; strains of *Sepedonium* spp. are kept in Laboratorio de Investigación en Agentes Antibacterianos, Universidad de Concepción, Chile and at KSH (Kulturen Sammlung Halle), Institute for Plant Biochemistry, Halle (Saale), Germany; voucher material (exsiccata of hosts infected by *Sepedonium* spp.) is deposited at Fungarium G. Palfner, Universidad de Concepción (CONC-F).

## Supplementary Materials

The following are available online at https://www.mdpi.com/article/10.3390/microorganisms9112261/s1, Table S1: Locations of the *Sepedonium* strains collected in Central Southern Chile with their respective hosts, Table S2: *Sepedonium* species/strains (in ascending alphabetical/numerical order) included in molecular phylogeny with their respective molecular markers and GenBank access numbers.
