# Supplementary material for: Diversity and Host Relationships of the Mycoparasite Sepedonium (Hypocreales, Ascomycota) in Temperate Central Chile
Source: Microorganisms. 2021 Oct 30;9(11):2261. doi: 10.3390/microorganisms9112261 (PMC8624339; doi:10.3390/microorganisms9112261)
Supplement: Supplementary file 1 [file microorganisms-09-02261-s001.zip › microorganisms-1394406-supplementary.pdf]

Binimelis et al. ***Sepedonium***

Supplementary material

Table S1: Locations of the *Sepedonium* strains collected in Central Southern Chile with their respective hosts.

Strain code	Species	Coordinates	Locality	Host
CONC-F1732	*S. ampullosporum*	LS 36° 49' 25,8''<br>LO 73° 00' 23,5''	Biobío Region, City of Concepción, private garden	*Paxillus involutus* under *Castanea sativa*
CONC-F1734	*S. ampullosporum*	LS 36° 52' 36''<br>LO 72° 59' 32''	Biobío Region, City of Concepción, Nonguén National Park	*Suillus luteus* under *Pinus radiata*
CONC-F1735	*S. ampullosporum*	LS 36° 52' 36''<br>LO 72° 59' 32''	Biobío Region, City of Concepción, Nonguén National Park	*Chalciporus piperatus* under *Pinus radiata*
CONC-F1736	*S. ampullosporum*	LS 36° 52' 36''<br>LO 72° 59' 32''	Biobío Region, City of Concepción, Nonguén National Park	*Suillus luteus* under *Pinus radiata*
CONC-F1741	*S. loyorum*	LS 38° 39' 18,59''<br>LO 72° 36' 11,43''	Araucanía Region, City of Temuco, Rucamanque Forest Reserve	*Gastroboletus valdivianus* under *Nothofagus dombeyi* and *N. obliqua*
CONC-F1743	*S. chrysospermum*	LS 38° 42' 7,57''<br>LO 72° 32' 57,38''	Araucanía Region, City of Temuco, campus Universidad Católica de Temuco	*Paxillus involutus* under *Nothofagus dombeyi*
CONC-F1744	*S. ampullosporum*	LS 36° 52' 36''<br>LO 72° 59' 32''	Biobío Region, City of Concepción, Nonguén National Park	*Suillus luteus* under *Pinus radiata*
CONC-F1752	*S. loyorum*	LS 38° 39' 20,06''<br>LO 72° 36' 13,07''	Araucanía Region, City of Temuco, Rucamanque Forest Reserve	*Boletus putidus* under *Nothofagus dombeyi* and *N. obliqua*
CONC-F1753	*S. loyorum*	LS 38° 39' 18,80''<br>LO 72° 36' 12,10''	Araucanía Region, City of Temuco, Rucamanque Forest Reserve	*Boletus loyo* under *Nothofagus dombeyi* and *N. obliqua*

CONC-F1754	*S. loyorum*	LS 38° 39' 7,09" LO 72° 36' 2,39"	Araucanía Region, City of Temuco, Rucamanque Forest Reserve	*Boletus putidus* under *Nothofagus dombeyi* and *N. obliqua*
CONC-F1755	*S. loyorum*	LS 38° 39' 18,79" LO 72° 36' 12,39"	Araucanía Region, City of Temuco, Rucamanque Forest Reserve	*Boletus loyo* under *Nothofagus dombeyi* and *N. obliqua*
CONC-F1756	*S. loyorum*	LS 37° 11' 47,62" LO 73° 00' 0,35"	Biobío Region, City of Santa Juana, native forest remnant	*Boletus putidus* under *Nothofagus obliqua*
CONC-F1758	*S. loyorum*	LS 38° 39' 25,76" LO 72° 36' 11,02"	Araucanía Region, City of Temuco, Rucamanque Forest Reserve	*Gastroboletus valdivianus* under *Nothofagus dombeyi* and *N. obliqua*
CONC-F1760	*S. loyorum*	LS 38° 39' 47,75" LO 72° 36' 25,93"	Araucanía Region, City of Temuco, Rucamanque Forest Reserve	*Boletus loyita* under *Nothofagus dombeyi* and *N. obliqua*
CONC-F1765	*S. loyorum*	LS 37° 48' 59" LO 72° 55' 30"	Araucanía Region, City of Angol, road to Nahuelbuta National Park	*Boletus loyo* under *N. obliqua*
CONC-F1766	*S. loyorum*	LS 37° 48' 59" LO 72° 55' 30"	Araucanía Region, City of Angol, road to Nahuelbuta National Park	*Boletus loyo* under *N. obliqua*
CONC-F1767	*S. chrysospermum*	LS 36° 52' 36" LO 72° 59' 32"	Biobío Region, City of Concepción, Nonguén National Park	*Suillus luteus* under *Pinus radiata*
CONC-F1768	*S. chrysospermum*	LS 36° 52' 36" LO 72° 59' 32"	Biobío Region, City of Concepción, Nonguén National Park	*Paxillus involutus* under *Pinus radiata*
CONC-F1769	*S. laevigatum*	LS 36° 52' 36" LO 72° 59' 32"	Biobío Region, City of Concepción, Nonguén National Park	*Suillus luteus* under *Pinus radiata*
CONC-F1777	*S. chrysospermum*	LS 38° 39' 29,77" LO 72° 36' 15,52"	Araucanía Region, City of Temuco, Rucamanque Forest Reserve	*Paxillus involutus* under *Nothofagus dombeyi* and *N. obliqua*

CONC-F1778	*S. chrysospermum*	LS 38° 39' 29,77" LO 72° 36' 15,52"	Araucanía Region, City of Temuco, Rucamanque Forest Reserve	*Paxillus involutus* under *Nothofagus dombeyi* and *N. obliqua*
CONC-F1779	*S. chrysospermum*	LS 38° 43' 38" LO 72° 35' 35"	Araucanía Region, City of Temuco, Ñielol Natural Monument	*Paxillus involutus* under *Nothofagus dombeyi* and *N. obliqua*
CONC-F1780	*S. loyorum*	LS 38° 39' 28,77" LO 72° 36' 11,76"	Araucanía Region, City of Temuco, Rucamanque Forest Reserve	*Boletus loyita* under *Nothofagus dombeyi* and *N. obliqua*
CONC-F1781	*S. chrysospermum*	LS 38° 27' 52" LO 72° 45' 50"	Araucanía Region, City of Temuco, Rucamanque Forest Reserve	*Paxillus involutus* under *Nothofagus dombeyi* and *N. obliqua*
CONC-F1786	*S. chrysospermum*	LS 38° 49' 49,5" LO 72° 02' 0,5"	Biobío Region, City of Concepción, campus Universidad de Concepción	*Paxillus involutus* under *Nothofagus dombeyi* and *N. obliqua*
CONC-F1821	*S. ampullosporum*	LS 37° 12' 39,5" LO 73° 33' 45,5"	Biobío Región, City of Arauco, Fundo Llico	*Suillus granulatus* under *Pinus radiata*
CONC-F1845	*S. chrysospermum*	LS 38° 39' 29,77" LO 72° 36' 15,52"	Araucanía Region, City of Temuco, Rucamanque Forest Reserve	*Paxillus involutus* under *Nothofagus dombeyi* and *N. obliqua*
CONC-F1846	*S. chrysospermum*	LS 38° 39' 29,77" LO 72° 36' 15,52"	Araucanía Region, City of Temuco, Rucamanque Forest Reserve	*Paxillus involutus* under *Nothofagus dombeyi* and *N. obliqua*
CONC-F1856	*S. loyorum*	LS 38° 39' 25,76" LO 72° 36' 11,02"	Araucanía Region, City of Temuco, Rucamanque Forest Reserve	*Gastroboletus valdivianus* under *Nothofagus dombeyi* and *N. obliqua*
CONC-F1857	*S. ampullosporum*	LS 36° 13' 27" LO 72° 37' 25"	Ñuble Región, City of Quirihue, Fundo El Guanaco	*Suillus luteus* under *Pinus radiata*
CONC-F1858	*S. ampullosporum*	LS 37° 22' 49,90" LO 73° 29' 9,11"	Biobío Región, City of Curanilahue, road to Villa Alegre	*Rhizopogon luteolus* under *Pinus radiata*

CONC-F1859	*S.ampullosporum*	LS 37° 22' 49,90" LO 73° 29' 9,11"	Biobío Región, City of Curanilahue, road to Villa Alegre	*Suillus luteus* under *Pinus radiata*
CONC-F1861	*S. ampullosporum*	LS 37° 25' 51,72" LO 73° 23' 48,62"	Biobío Región, City of Curanilahue, road to Huillinco	*Suillus luteus* under *Pinus radiata*
CONC-F1867	*S. laevigatum*	LS 36° 52' 42" LO 72° 59' 33"	Biobío Region, City of Concepción, Nonguén National Park	*Rhizopogon roseolus* under *Pinus radiata*
CONC-F1869	*S. ampullosporum*	LS 38° 39' 29,77" LO 72° 36' 15,52"	Araucanía Region, City of Temuco, Rucamanque Forest Reserve	*Suillus luteus* under *Pinus radiata*

Table S2: *Sepedonium* species/strains (in ascending alphabetical/ numerical order) and outgroups included in molecular phylogeny with their respective molecular markers and GenBank access numbers. CONC-F strain code sequences were generated in this study; missing sequences (-) were not available in GenBank and/or could not be obtained through the applied PCR protocol.

		**DNA markers and GenBank access numbers**		
**Species**	**Strain code**	**ITS1**	**EF1-α**	**RPB2**
S. ampullosporum	CONC-F1732	MW386316	MZ285598	MZ360964
S. ampullosporum	CONC-F1734	MW386317	-	-
S. ampullosporum	CONC-F1735	MW386318	MZ308648	MZ360965
S. ampullosporum	CONC-F1736	MW386339	MZ360967	MZ360966
S. ampullosporum	CONC-F1744	MW386486	-	-
S. ampullosporum	CONC-F1821	MW386488	MZ343800	MZ366757
S. ampullosporum	CONC-F1857	MW386489	MZ343801	MZ389106
S. ampullosporum	CONC-F1858	MW386493	MZ343802	MZ389107
S. ampullosporum	CONC-F1859	MW390916	MZ343803	MZ389108
S. ampullosporum	CONC-F1861	MW386627	MZ333232	MZ389109
S. ampullosporum	CBS645.66	MH858903.1	-	-
S. ampullosporum	S15	AF054853.1	-	-

S. ampullosporum	WIC 001	MG052636.1	-	-
S. ampullosporum	WIC 007	MG052634.1	-	-
S. brunneum	AF054861.1	AF054861.1	-	-
S. chalcipori	CBS148.92	MH862347.1	-	-
S. chalcipori	CBS168.92	MH862349.1	-	-
S. chalcipori	CBS278.92	MH874023.1	-	-
S. chalcipori	JAC14133	MK432800.1	-	-
S. chalcipori	KSH 401	KT946840.1	KU041502.1	KU041490.1
S. chalcipori	KSH 587	KT946846.1	KU 041508.1	KU041496.1
S. chalcipori	KSH 558	KT946845.1	KU041507.1	KU041495.1
S. chalcipori	KSH 602	KT946847.1	KU041509.1	KU041497.1
S. chalcipori	S32	AF054864.1	-	-
S. chalcipori	S33	AF054863.1	-	-
S. chalcipori	S35	KT946840.1	-	-
S. chalcipori	S44	KT946846.1	-	-
S. chlorinum	AF54866.1	AF054866.1	-	-
S. chrysospermum	CONC-F1743	MZ272442	MZ333230	MZ366750
S. chrysospermum	CONC-F1768	MZ272474	MZ333231	MZ366753
S. chrysospermum	CONC-F1777	MZ272476	MZ322966	MZ366755
S. chrysospermum	CONC-F1779	MZ272477	-	-
S. chrysospermum	CONC-F1781	MZ292899	MZ329802	MZ366756
S. chrysospermum	CONC-F1786	MZ272698	-	-
S. chrysospermum	CONC-F1845	MZ272885	MZ329801	MZ389105
S. chrysospermum	CONC-F1846	MZ273040	-	-
S. chrysospermum	S47	AF054844.1	-	-
S. laevigatum	CONC-F1769	MW386487	MZ333233	MZ366754

S. laevigatum	CONC-F1867	MW386632	MZ333234	MZ389110
S. laevigatum	CBS101645	NR119422.1	-	-
S. laevigatum	S45	AF054856.1	-	-
S. laevigatum	S88	AF054857.1	-	-
S. laevigatum	S173	AF054858.1	-	-
S. laevigatum	S176	AF054855.1	-	-
S. loyorum	CONC-F1741	MW396890	-	-
S. loyorum	CONC-F1753	MW386647	MZ343804	MZ389111
S. loyorum	CONC-F1754	MW386648	MZ343805	MZ389112
S. loyorum	CONC-F1755	MW386649	-	-
S. loyorum	CONC-F1760	MW396888	MZ343806	MZ366751
S. loyorum	CONC-F1765	MW386650	MW759845	MZ366752
S. loyorum	CONC-F1766	MW386651	-	-
S. loyorum	CONC-F1780	MW396889	-	-
S. loyorum	CONC-F1856	MW396891	-	-
S. loyorum	KSH 883	KT946848.1	KU041510.1	KU041498.1
S. loyorum	KSH 928	KT946850.1	KU 041512.1	KU041500.1
S. microspermum	S6	AF054849.1		
S. microspermum	S23	AF054851.1	-	-
S. microspermum	S24	AF054847.1	-	-
S. microspermum	S77	AF054848.1	-	-
S. microspermum	S155	AF054850.1	-	-
S. microspermum	S177	AF054852.1	-	-
S. tulasneanum	CBS940.69	MH871270.1	-	-
S. tulasneanum	S29	AF054859.1	-	-
S. tulasneanum	S165	AF054860.1	-	-

Cladobotryum heterosporum	CBS 719.88	NR111428.1	-	-
Cladobotryum paravirescens	TFC 97-23	NR121424.1	-	-
Cladobotryum tchimbelense	TFC 201146	TFC 201146	-	-
Hypomyces australasiaticus	TFC 03-8	NR121428.1	-	-
Hypomyces gabonensis	TFC 201156	NR121429.1	-	-
Hypomyces samuelsii	CBS 127157	NR121403.1	-	-
Trichoderma aerugineum	FJ860516.1	-	-	FJ860516.1
Trichoderma aerugineum	FJ860608.1	-	FJ860608.1	-
Trichoderma aerugineum	NR134379.1	NR 134379.1	-	-
